# Engaging Honduran Science Diasporas for Development: Evidence From Three Consolidated Networks

**DOI:** 10.3389/frma.2022.899631

**Published:** 2022-06-16

**Authors:** Kleinsy Bonilla, Karina Aquino Valle, Ramon Alvarez-Torres, Sara Ney Simons

**Affiliations:** ^1^Department of Science and Technology Policies, Institute of Geosciences, State University of Campinas, Campinas, Brazil; ^2^Instituto para el Desarrollo de la Educación Superior en Guatemala (INDESGUA), Guatemala City, Guatemala; ^3^Organization for Women in Science for the Developing World, Trieste, Italy; ^4^University Scientific Research Office, National Autonomous University of Honduras, Tegucigalpa, Honduras; ^5^Department of Sociology, National Autonomous University of Honduras, Tegucigalpa, Honduras

**Keywords:** science diaspora, Honduras, S&T policy, S&T capacity building, Central America, Honduras Global, OWSD, Escuela Zamorano

## Abstract

Honduras' underdevelopment of the higher education system, national economic constraints, and low investment in science and technology (S&T) have created significant challenges in training, employing, and retaining its science workforce, resulting in what is known as “brain drain” in literature. There are no official statistics of Honduran scientists who have established their residency abroad, nor the Honduran scientific diasporas (HSD); however, various diaspora networks provide evidence of their existence and engagement in their home country. This study takes an empirical approach and explores experiences of networking and engagement of the HSD for the development of Honduras. Methodologically, a qualitative approach and a phenomenological design were used. The data were collected through documentary review and semi-structured interviews with 21 key respondents from three identified HSD networks: Honduras Global (HG), the Organization of Women in Science for the Developing World, Honduras National Chapter (OWSD Honduras), and the Alumni Association of the Zamorano Pan-American Agricultural School (AGEAP-Zamorano). The holistic analysis of HSD's engagement provides evidence of existing registry gaps. Neither the S&T agents nor the Honduras Foreign Policy have identified, mapped, and characterized Honduran scientists' emigration patterns. Evidence suggests the willingness of the HSD to transfer knowledge, build bridges, and facilitate access to world-class research practices to their peers residing in Honduras and interact with broader sectors of the Honduran society.

## Introduction

For decades, the international mobility of scientists from the Global South to the North has been the subject of several fields (Barré et al., 2013; Geuna, 2015). Such mobility has been less examined when considering the establishment of permanent residence of such scientific workforce in advanced countries (long-term immigration) as opposed to quick schemes of mobility (i.e., participation in training and short-term educational programs). As the global competition to attract highly educated individuals has intensified, the emergence of phenomena, known as “brain drain” (Durmaz, 2020), “human capital flight” (Popogbe and Oluyemi, 2020), and “academic exodus” (Heffernan and Heffernan, 2020), has dominated the literature. These concepts assume the loss of talented and outstanding professionals who flee from one country or region (developing) instead of another (developed). However, new approaches propose a paradigm shift toward “brain circulation” (Fangmeng, 2016), “knowledge diaspora networks” (Meyer, 2011), and “expatriate scientists” (Barré et al., 2013), addressing the formation of science diasporas (SD) from a more optimistic perspective. The scientific and technology diasporas (S&T diasporas) have been defined as “self-organized communities of expatriate scientists and engineers working to develop their home country or region, mainly in science, technology, and education” (Barré et al., 2013, p. 83). In other words, the S&T diasporas are formed by highly skilled scientists, researchers, and engineers, who live, work, and reside in countries other than their nation of origin. Until recently, the most vocal concern pointed to the occurrence of brain drain; however, in the digital area, instead of insisting on the return of the highly skilled migrants, the emphasis is placed on the circular exchange and transnational mobility (Stopić, 2013). From a global perspective, it is essential to focus on the interlocking webs of international and national development organizations, international organizations, public institutions, and migrant associations and networks. These provide a roadmap for this wide-ranging terrain of structural transformation. In this context, it is helpful to think about migration and development in a transnational circulation, placing the migrant at the center of attention, and identifying them as a cooperation and development agent (Meyer, 2011). Notwithstanding, the emphasis is placed on the role of financial remittances. The activities of organizations and the positioning of agents within networks constitute a social transformation work; in this way, engaging with the diaspora for the benefit of development has thus become an essential strategy of many immigration states.

The role of diasporas in development strategies, poverty reduction, and economic growth is attracting considerable policy interest, involving diasporas, host countries, and home countries (Ionescu, 2006). Barré et al. (2013) mention that some basic S&T diasporas' activities are “building a scientific community, gathering, managing, and circulating information about members' skills, organizing scientific events and training, and contributing to S&T infrastructure in the home country” (p. 102). These diasporas are special agents for developing in Northern countries issues specific to the scientific agenda of the South. It is advantageous that these S&T diasporas have a clear understanding of the reality and problems faced in their countries of origin which in turn facilitate transnational interaction. Barré et al. (2013) studied various cases involving Latin American SD: one on the experience of non-profit organizations (NGO) in France (AFUDEST and ALAS), one on work in an international organization (UNESCO), and one about fieldwork with a government agency, Argentina's Secretariat for Science, Technology and Productive Innovation (SETCIP) (p. 406).

In Central America, the dominant paradigm is also “brain drain.” In this region, the prevalent perception is that the lack of career opportunities and low S&T national capacities make it challenging to retain highly skilled and well-educated scientists and researchers (Bonilla, 2022). For this reason, accomplished scholars decide to establish their residence in other countries where the labor condition can meet their goals. Admittedly, the S&T ecosystems at the global, regional, and national levels in the digital age are experiencing incentives to turn brain drain into brain circulation, considering that these two concepts have been running long in the scholarly debate (Stopić, 2013). While accepting that scientific diasporas from countries in the Global South exist, a key question emerges: What experiences of engagement of such SD with their country of origin exist and which lessons can be extracted? This research attempted to delve into this question focused on the case of Honduras.

As a Central American country, Honduras is a scientifically lagging country, sharing the same obstacles as the region to keep their highly skilled professionals. Admittedly, according to Discua and Cerrato (2019), the country has started to recognize prominent Hondurans in arts, entrepreneurship, international business (Discua and Cerrato, 2019), and social enterprise of the Honduran diaspora (Discua and Fromm, 2018). As for science diasporas, there is no official or centralized database of the HSD built by any public institution (i.e., Ministry of Foreign Affairs, Secretariat of Planning, Honduras Institute of Science and Technology) in Honduras. Therefore, the authors applied an exploratory search for SD networks related to higher education, international cooperation, and skilled migration from Honduras. This is how three diaspora networks were identified: (i) Honduras Global, (ii) The Organization of Women in Science for the Developing World-OWSD Honduras National Chapter (OWSD Honduras), and (iii) Association of Alumni from the Pan-American School of Agriculture Zamorano School (AGEAP-Zamorano) (Details are presented in section Methods, Table 1). This study aimed to understand in depth the engagement experiences of the Honduran science diaspora (HSD) for the development of Honduras in the 2010–2022 period, specifically the HSD of the three mentioned networks. The produced knowledge in this research will help continue the conceptual and empirical development of the alternative optimistic perspective to migration outflows, emphasizing the circular exchange and transnational mobility in the migration–development nexus. Also, understanding the engagement experiences will offer feedback to improve and continue the work in this field because this is the first systematization in the country on this topic. The engagement is approached from an adaptation of the typology included in the theory of participation of the stakeholder and public engagement proposed by Reed et al. (2018). This is based on the agency (who initiates and leads engagement) and mode of engagement (from communication to co-production). They define participation:

as a process where public or stakeholder individuals, groups, and organizations are involved in making decisions that affect them, whether passively via consultation or actively via two-way engagement, where publics are defined as groups of people who are not affected by or able to affect decisions but who engage with the issues to which decisions pertain through discussion and stakeholders are defined as those who are affected by or can affect a decision. (p. 2).

**Table 1:** Honduras diaspora engagement for development.

**Type of engagement**	**Categories and operationalization**	**Operationalization/illustrative examples**
Orientation	Top-Down: initiated and/or led by those with formal decision-making power who wish to empower interested parties with less power and diverse perspectives to make or contribute toward decisions	Approach in which an executive decision maker or other top person makes the decisions of how something should be done. This approach is disseminated under their authority to lower levels in the hierarchy, who are, to a greater or lesser extent, bound by them. Public policies, legislative actions/programs, guidelines.
	Bottom-Up: initiated and/or led by citizen, public or special interest groups with limited formal decision-making power	Approach in which is the piecing together of systems to give rise to more complex systems, thus making the original systems subsystems of the emergent system.
Direction	Unidirectional: Communication, Consultation	Experiences with passive audiences: e. g.., podcasts, webinars, scientific dissemination, presentations, online teaching
	Bidirectional: Collaboration between two parts	Experiences involving active exchange and co-creation between two clearly identified parties: e.g., Thesis review, project evaluation, research extension
	Multidirectional: Collaboration between multiple parts	Experiences with higher complexity with active exchange and co-creation involving clearly identified multiple parties. International projects collaborations with the participation of consortiums

*Source: Adapted from Reed et al. (2018, p. 31)*.

Considering the descriptive typology that attempts to explain the outcomes of engagement in any given context, Table 1 shows the adaptation of the types of engagement of the SD as a guiding framework for this research.

## Scope and Limitations

A few limitations of the study must be acknowledged. Due to the lack of comprehensive databases of the HSD, results and findings are not statistically relevant. We instead focused on collecting and analyzing rich qualitative data to extract learnings from the experiences. In addition, the potential interviewees share a common characteristic: limited time available to participate in the study. At first, early and mid-career members of the HSD were more prone to experience. Therefore, we needed to run a complementary invitation to ensure further participation of established-career members. Finally, the temporal delimitation is relatively short (12 years); this obeys the characteristics of the HSD networks. Although AGEAP-Zamorano can be traced back a few decades of existence, it was just after the turn of the 2000s when the networking component took hold. HG was launched in 2011, while OWSD Honduras was launched in 2020.

## Methods

Methodologically, the object of study was approached through qualitative research and a phenomenological design (Creswell and Poth, 2018). Regarding temporality, information has been collected since the origin of two HSD networks: Honduras Global 2011–2022 OWSD Honduras 2021–2022. In the case of AGEAP-Zamorano, the availability of updated records delimited the period 2010–2022. Two sources of data were used: documentary review and semi-structured interviews. The documentary review covers files on the experiences in Honduras involving the HSD, including historical reports, institutional annual reports, strategic plans, annual operating plans, press publications with reports, audiovisual material, journalistic notes, and media coverage of the initiatives. In the second, the population of participants included members of the HSD belonging to the three mentioned networks; Table 2 summarizes their characteristics.

**Table 2:** Honduras scientific diaspora selected networks.

**HSD Network**	**Characteristics**
Honduras Global^*^	Honduras Global is a Foundation launched in 2011, inspired by the international network of “outstanding” Hondurans promoted by Sir Salvador Moncada, a prominent scientist with roots in Honduras based in the United Kingdom. As of February 2022, it has over 60 members, including artists, entrepreneurs, businesspeople, and scientists. All its members are Hondurans.
OWSD Honduras^**^	OWSD Honduras National Chapter is a community of Honduran women scientists formally established in July 2021. As of February 2022, it has 97 members, from which 29 report their place of residence and work abroad.
Zamorano Alumni^***^	The Association of Zamorano Alumni is a systematic networking platform established in 1965. As for 2022, there are nearly 9,000 graduates from over 30 countries of origin. The Alumni is organized in chapters based on their location, interests and affiliations, e.g., There are Alumni Zamorano Association in Europe, the United States, Asia, Africa and various countries in Latin America

* *Database based on http://hondurasglobal.org/*.

** *Database based on http://owsd.net/network/honduras*.

*** *Alumni Zamorano (AGEAP): Asociación de Graduados Escuela Agrícola Panamericana based on https://www.zamorano.edu/graduados*.

The research technique chosen for this research was the semi-structured interview. This methodological tool is characterized by deciding in advance the type of information required, and based on the objectives, a script of questions is created; unlike structured interviews, these have the particularity of being more flexible concerning the order, priorities, or requirement of deepening (Bertomeu, 2016). This type of interview was chosen as it intends through the collection of a set of private knowledge, the construction of the social meaning of individual behavior, or the reference group of the interviewed subject, in this case, the HSD. Likewise, this type of interview facilitates the collection and analysis of social knowledge crystallized in discourses, which have been constructed by the direct and unmediated practice of the protagonists; therefore, it allows us to have a first approach to a topic that is largely unexplored in the country.

The inclusion criteria for the participants in the semi-structured interviews were designed to procure diversity in the representation of fields of knowledge, geographic location of residence among HSD, and balance in gender participation. The sampling was purposeful of the homogeneous and chain type (Creswell, 2015; Creswell and Poth, 2018). We established contact with an official representative of the networks. We visited their websites to obtain a list of members with their general characteristics, then selected the possible participants according to the inclusion criteria, and thus, were invited by email. When the interviews were done, we asked for recommendations from other participants. The profiles of the participants are summarized in Table 3.

**Table 3:** Key respondents semi-structured interviews HCD selected platforms.

**HCD network**	**Code**	**Experience**	**Trajectory**	**Destination**	**Gender equity**	**Field of expertise**
Honduras Global (HG)	HG1	Researcher and Scholar	Established Career	Bern/ Switzerland	F	Agricultural Sciences/Sustainable Development
	HG2	Researcher and Bioinformatician	Early Career	Canada/ British Columbia/	M	Health Sciences/Cancer Epigenomics
	HG3	Postdoctoral Researcher	Mid-Career	Denmark/ Odense	M	Food Sciences/Microscopic Composition
	HG4	Researcher, Senior Lecturer	Established Career	United Kingdom/ Lancaster	M	Business Research/Family Businesses
	HG5	Graduate Student Doctoral Program	Early Career	The Netherlands/ Amsterdam	F	Health Sciences/Epidemiology
	HG6	Graduate Student Doctoral Program	Early Career	France/ Paris	M	Health Sciences/Virology
	HG7	Senior Researcher	Established Career	Belgium/ Vrijes	M	Psychology/Neurosciences—Emotions
OWSD Honduras (OWSD-NH)	OWSD-HN1	Graduate Student Doctoral Program	Early Career	Spain/ Valencia	F	Environmental Sciences/Geographic Information Systems
	OWSD-HN2	Graduate Student Doctoral Program	Early Career	United States/ Washington	F	Mathematics/Computational Mathematics
	OWSD-HN3	Postdoctoral Researcher	Mid-Career	Mexico/ Merida	F	Organisms and Biological Systems/Plant Molecular Biology
	OWSD—HN4	Researcher Industry	Mid-Career	Spain/ Valencia	F	Chemical Engineering/Nanotechnology
	OWSD—HN5	Graduate Student Doctoral Program	Early Career	Mexico/ Mexico City	F	Economic and Financial Sciences /Social Innovation and Social Responsibility
	OWSD—HN6	Senior Scholar	Established Career	Germany/ Kaiserslautern	F	Organisms and Biological Systems/Environmental Change
	OWSD—HN7	Senior Researcher	Established Career	United States/ California	F	Health Sciences/epilepsy Neurosciences
Zamorano Alumni Association (AGEAP)	AGEAP1	Graduate Student Master's Program	Early Career	The Netherlands/ Gelderland	M	Food Sciences and Technology/Food Chemistry
	AGEAP2	Graduate Student Master's Program	Early Career	USA/ Miami	F	Livestock Sciences/Genetics
	AGEAP3	Graduate Student Doctoral Program	Early Career	USA/ Louisiana	M	Nutrition and Food Sciences/Food Innovation
	AGEAP4	Graduate Student Doctoral Program	Early Career	USA/ Alabama	F	Poultry Sciences/Infrastructure
	AGEAP5	Associate Professor	Established Career	USA/ Texas	M	Food and Resource Economics/Sustainable production
	AGEAP6	Project Officer	Established Career	Switzerland/ Lausanne	M	Environmental Engineering/Risk Management
	AGEAP7	Senior Researcher and Consultant	Established Career	Colombia/ Bogota	M	Agricultural Sciences/Soil yield and bioproducts

*N = 21; F, = Female (N = 11); M, = Male (N = 10)*.

## Discussion and Findings

### Integration of the Honduran Scientific Diasporas (HSD)

The understanding of the participants of the concept of “scientific diaspora” varies significantly. While most of them did not have an in-depth knowledge of the term, many associate it with migration and international mobility. Nearly all the participants related the term scientific diaspora to “brain drain.” Traditionally, this connotation has a negative view of the consequences of subtracting highly qualified people from their place of origin in the workforce.

In this sense, AGEAP2 points out the following: “the only thought that comes to my mind when I hear “scientific diaspora” […] (is) brain drain. […] I feel my country loses this valuable talent due to the current situation in Honduras and the few opportunities for us to work there as scientists, especially the young people.” OWSD-ND agrees and adds: “the term [HSD] suggests the presence of Hondurans deployed in other universities around the world; this one is also a sign of the brain drain which at the same time speaks highly of the scientific capacity that we have in Honduras.” It is important to emphasize that participants referring to the situation of lack of opportunities tended to focus more on the reasons for the departure than on the possible damage that their country of origin could face due to the release of large numbers of skilled human capital. Another recurrent association of terms was diasporas in emigration. Yet, when participants consider themselves as migrants, some of them point out apparent differences from the vulnerable migration Honduras has experienced for decades. HG1 and HG2 agreed that emigrating with proper documentation (visa, valid passport) and institutional support marks a different path toward relocation compared with the precariousness of irregular (economic) emigration from Honduras to the United States of America. In this sense, other terms loaded with more positive perspectives are expatriates, skilled migrants, and international scholars. The value assigned to the term “scientific diaspora” remains relatively positive or neutral for most participants. Such is the case of OWSD-HN3, who states: “[as SD] When I think of seeds, I think of a group of people who come from the same place and are dispersed elsewhere,” OWSD-HN6 concurs: “my background as biologist gives me notion [of diaspora] as seeds planted in various places doing science and research.”

Another association to SD was the expressed desire to remain linked to their country of origin and contribute to improving the Honduran population's lives. HG4 noted the following “for one reason or another, we live outside our country, but we maintain a very closely linked with what is happening there and want to contribute to improve things.” In repeated interventions, participants emphasized their desire to “contribute” or “give back to the country,” especially when they have benefited with scholarships or funding based on their nationality. These attitudes are consolidated from the exposures the HSD have to better research practice in their country of destination, which gives them an awareness of the various science gap existing with Honduras, for example, infrastructure, human power, and institutional support. AGEAP6 and HG7 gave similar statements.

As for the motivations for organizing and belonging to their respective HSD networks, it was found that most participants report three levels of reasons: personal, professional, and organizational. Participants listed among their motivations as building win–win scenarios. They benefit in their career development while also offering their time, knowledge, skills, and contacts to carry on further reaching activities. Joining a community where they interact with peers and actors from other sectors is part of their objectives. Participant OWSD-HN1 indicated: “I felt this need to interact with colleagues, share interests, and build alliances.”

On the contrary, among the people whose motivation relates to organizational aspects, it can be observed that most participants have altruistic motivations and find their networks enable them to pursue their goals. They sought to share the knowledge acquired abroad, either with vulnerable populations or with their peers. Such was the case of OWSD-HN3, who declared: “[my motivation to be part of my network is] to disseminate science results and engage in teaching so that it can encourage girls for them to study in STEM [science, technology, engineering, mathematics] careers.” The goal that various members of the HSD recurrently pointed out was exercising influence to improve the living conditions in Honduras.

The HSD shows a concentration in two geographic destinations, the United States and Europe. For a long time, the United States has been perceived as a land of economic opportunity by Hondurans (OAS-IDB, 2021), who for decades have singled out this country in North America as a priority destination, mainly for economic migration. This has created bonds and migration flows from Honduras, also found in skilled migration. This is partly explained by geographic proximity and the notoriety of American universities in Honduras. Another factor, explaining the concentration of the HSD in the United States, Canada, and Europe, is the provision of scholarships for training at master's and doctoral levels, for which Honduran citizens have been eligible for decades. In this sense, other countries of destinations that also support Honduran graduate students (notably Taiwan and South Korea) have engaged in cooperation with Honduras in the last decade. Therefore, the presence of HSD in Asian countries seems to be more limited. Complementarily, participants from the three HSD networks highlighted the overwhelming concentration of its members in North America and Europe.

### Engaging the HSD (Types of Engagement)

In analyzing the types of engagement involving the HSD, two categories guide the presentation of the main findings. The first elaborates on the types of engagement in the dichotomy Top-down/Bottom-up approach, and the second addresses the types of engagement according to the direction of the interactions: unidirectional, bi-directorial, and multidirectional.

#### Orientation of the Engagement: Top–Down/Bottom–Up Approach

The orientation of the engagement refers, on the one hand, to the Top-down approach in two main lines: the Engagement of the HSD promoted by (i) S&T policy agents and (ii) Foreign policy. These two cases involve the actions emanating from an authority. On the other hand, the Bottom-up approach refers to initiatives promoted by individuals, which evolve into networks, going from part to a system.

##### The HSD and the S&T Policy Agents

In Honduras, at the governmental level, the official development of science has been recent (limited progress in the last 30 years) and has not had consistency between the changes in government administration. Science has been given little priority and little funding, and no national plan has been published to date. Honduras has structured the National System of Science, Technology, and Innovation (SNCTI). Few government entities make up this system, lacking the integration with other actors, including universities, companies, non-governmental organizations, and civil society organizations. In this sense, no legal and officially expressed articulation forms a comprehensive and multisectoral system. In the light of this, the scientists in the majority reported that they have not heard about government initiatives that articulate the scientific diaspora in Honduras. They mentioned some initiatives that promote scholarships, such as *Becas 2020*, which encourages Honduran students to continue their studies abroad under the condition of returning to the country. The recognized Alumni Zamorano network, except for *Becas 2020*, informed us about HONDUFUTURO, a private institution that finances 50% of the scholarship (AGEAP 1).

The government barely promotes scientific initiatives, and it makes sense with the lack of information the researcher reported. The main question is, how could they return to a country that does not provide the minimum conditions to create opportunities to research and overcome the brain drain. In the digital era, where virtuality is an essential element of development, the “brain circulation” of scientists of the Global South could increase the sustainable development of their countries, taking advantage of the resources and networks of their residency countries. OWSD-HN5 states that the “government should take advantage of my new knowledge produced by Hondurans. Still, we generated this disconnection with researchers residing in other countries that could be used for the development of Honduras.” Several participants mentioned that consecutive governments of Honduras had few public institutions interacting with researchers and scientists in general, let alone those nationals from Honduras living abroad. AGEAP2 uses the example of the Direction for Innovation and Technology (DICTA), which has not fulfilled its role as a public research institution; no scientists work on researchers' projects there. DICTA employs officers with inadequate backgrounds; there is no STI (science, technology, and innovation) functional ecosystem that provides conditions to Honduran researchers. AGEAP 3 mentioned that “every Honduran would like to contribute directly to the development and return to their home living in Honduras. However, it seems that the governments are not interested in engaging us.” AGEAP7 provided a practical action taken by a Honduran public institution. They who acknowledge the critical involvement of the *Secretaria Técnica de Planificación y Cooperación Técnica* (SEPLAN^1^). in the partnership which resulted in the creation of *Honduras Global* (HG) in 2011. This initiative—HG—is referred to by most of the participants in this study as the most known platform to engage the HSD in the development of Honduras.

##### HSD and the Honduras Foreign Policy

Honduras's foreign policy concerning S&T has been practically reduced to administering international cooperation to educate and train promising young researchers. In other words, they were handling fellowships and scholarships to train graduate students in master's and doctoral programs in international universities. Yet, various governmental institutions, including the Ministry of Foreign Affairs, lacked systematic and transparent practices (Bonilla and Serafim, 2021). The connection between the scientific diaspora within mechanisms to contribute to tackling the issues of their country of origin, in the case of Honduras, has not been explored. According to Balakhrisnan (2018), Diplomacy for Science is understood as promoting international science cooperation, a dimension of Science Diplomacy that could create evidence-based public policies if this goes in line with government priorities. However, most interviewed members of the HSD indicated interactions with the embassies, consulates, and diplomatic missions of Honduras accredited in their countries/cities of destination regarded only to migration-related procedures (e.g., renewal of passport, emission of the national identity card), with not a single mention of purposeful engagement in their capacities as scientists. They reported no interest in the embassies trying to connect them to the country. HG3 explained “concerns about [inexistent] channels to connect the HSD with scientific projects in Honduras because of the lack of interest from the Embassies abroad.”

The foreign policy of Honduras has focused mainly on reducing the forced-irregular emigration to the United States of America. According to Meyer (2022), over the 2010–2020 decade, a pervasive combination of factors has triggered the dramatic phenomena of the Caravan of Migrants, in which Honduras (along with Guatemala and El Salvador) has expelled their citizens. Such factors include violence and repeated droughts linked to climate change that has increased food insecurity, particularly for subsistence farmers in the Dry Corridor of Central America.

In Central America, governments, academia, and the private sector increasingly recognize the importance of science, technology, and innovation (STI) as drivers of long-term, sustainable growth (Padilla Pérez, 2013). In the same way, the government of Honduras recognizes the importance of promoting, guiding, and encouraging scientific, technological, and innovation advancement to formulate medium and long-term plans. However, there is a lack of communication with scientific communities and the policymakers, despite the country leading on a legal basis and proper institutional framework supported for the parameters given by the international cooperation system. Science Diplomacy plays an important role that bridges both arenas of science and diplomacy to face global challenges. Due to emphasis on the term, science diplomacy is the use of scientific collaborations among nations to address the common problems confronting twenty-first-century humanity and build constructive international partnerships (Fedoroff, 2009). One of the approaches of science diplomacy addresses the importance of scientific networks informing public policies. Scientific communities play a vital role in advancing and updating knowledge in the region, promoting strategies for governments, universities, research centers, and civil society. Mainly, these strategies seek to work on Central America's integration of knowledge to encourage exchange, capacity building, and high-level training in the region. At this point, Honduras lacks this expertise; however, the challenge ahead is to build on the promising first steps and to enhance the contribution of research to sustainable development.

##### Bottom–Up Engagement to the HSD (Honduras Global, OWSD Honduras, Alumni Zamorano)

In the absence of government support, networks and organizations based on individual connections, such as Honduras Global, OWSD Honduras, and Alumni AGEAP-Zamorano, have played a significant role in articulating and engaging the HSD with their country of origin. The referred networks have taken steps forwards in achieving their objectives. Various participants offered examples of other networks organized around scholarship programs such as Fulbright (the United States of America), DAAD (Germany), Chevening (United Kingdom), and MASHAV (Israel), among others. However, they also pointed out that the level of engagement involved mainly dissemination of the scholarships and promotion of the partner countries with limited attention to the root development challenges of Honduras.

##### Honduras Global

Honduras Global^2^ is a foundation created in 2011 and promoted by Sir Salvador Moncada. Its foundational objective focused on identifying and connecting highly skilled and prominent Hondurans located in different countries and regions worldwide to facilitate the transfer of knowledge and talents and promote innovation and scientific, technological, and business development in Honduras. At the launching of the initiative, cooperation from the public sector (Secretary of Planning) and international partners (GiZ from Germany) provided support. Among its principal activities, its members organize and implement science dissemination events (podcasts, presentations, interviews) and collective events such as the Week of Science, which had various editions, until the COVID19 pandemic in which it was suspended since 2020. As of February 2022, it registered 49 associates with networks geographically active in North America and Europe.

##### OWSD Honduras

The OWSD Honduras^3^ is the national section of the global Organization of Women in Science for the Developing World. It was established in October 2020 and is based in Tegucigalpa. OWSD as a worldwide organization has existed since 1989 as a unit of UNESCO; however, in the case of Latin America, it was not until 2019 that the first national section was created. OWSD Honduras was the fifth in the Latin American and the Caribbean region. Among the principal activities are creating a repository for identifying Honduran women scientists and their respective areas of study, disseminating scientific awareness through webinars, workshops, and seminars for the scientific community and the public, and organizing leadership training for women in STEM. A component of this network is incorporating Honduran women scientists residing overseas. As of February 2022, OWSD Honduras had 97 members, of which nearly 30% (30) reported their place of residence abroad.

##### Alumni Zamorano-AGEAP

The Pan-American School of Agriculture Zamorano was created in 1942 in Honduras as a technical-oriented educational project in agriculture and agribusiness. Since then, it has graduated over 9,000 alumni from 30 countries. The school has achieved regional recognition for its emphasis on leadership and commitment to developing its students' countries of origin. In 1965, the Zamorano Alumni Association^4^ (AGEAP is an acronym in Spanish for Asociación de Graduados de la Escuela Agrícola Panamericana). This network is structured in regional and national chapters in which its members actively engage in interactions among themselves and organize and participate in activities related to Honduras. Notably, active chapters in which Hondurans participate include Asia–Africa, Europe, the United States of America (a significant share of members have their residence in the USA), and other countries in Latin America. The main activities in which AGEAP-Zamorano participates emphasize self-development and career progression; however, they also participate in communication and dissemination activities targeting broader sectors of the Honduran society back in their country of origin.

#### Direction of the Engagement: Unidirectional–Bidirectional–Multidirectional

The types of engagement based on the direction of the interactions have been categorized in this research in three pathways: (i) Unidirectional (when the engagement activities involve an active agent, in this case, the member of the HSD and a passive audience), (ii) bi-directional (the engagement includes co-creation in the collaborative activities from at least two parties, in general, linking the HSD with peer scientists, students, and other actors connecting country of destination and country of origin), and (iii) multidirectional (the engagement enable complex interactions among parties in multiple locations).

Most activities in which the participants have engaged classify in the first category: unidirectional. Members of the HSD are in their early and mid-careers, and they commonly participate in science communication and dissemination activities, for example, webinars, fora, mentoring, and podcasts (OWSD-HN1, OWSD-HN4, and OWSD-HN5), teaching and training young students (OWSD-HN5 and OWSD-HN3). Once the HSD moves toward further career development, participation in bi-directional engagement arises. HG1 and AGEAP5 mentioned their contributions in academic exchanges and joint publications with peers working in Honduras. Likewise, HG2 and AGEAP4 said they collaborate in research among the members. Some scientists go beyond and explore how to contribute to the country's main challenges. For example, HG1 and OWSD-HN3 have developed initiatives involving multiple parties from various countries.

While HG1 promoted visits from Honduran students to various research facilities in European countries, OWSD-NN3 reported participating in a research project collaborating with the members of Alumni Zamorano, her *Alma Mater* in Honduras. AGEAP6 shares a clarifying example: “as I gained more seniority in my career, I was able to mobilize various millions of dollars in projects to be implemented by my affiliated institution in Europe in partnership with Honduran organizations back in my home country in fields related to climate change and environmental vulnerability.” In the same way, other participants provided examples of developing projects on water and sanitation issues for vulnerable Honduran communities. An essential element has been found that strongly engages the HSD aims to close the inequality prevalent in Honduras. OWSD-HN2 stated, “I feel like an agent of change and on behalf of black women in Honduras who do not have many spaces won in education, mathematics, science, engineering, and many more.” She advocates for minorities. These multidisciplinary female scientists know the social and economic challenges of the country very well, even more than the government is aware of, and they seek activities that are based on tackling these issues to contribute to sustainable development. AGEAP3 has been inquiring about participating in volunteer programs to create scientific capacity in government institutions without reaching good results. OWSD-NH7 shares: “My experience was coordinating a collaborative project of national coverage; this enabled scientific publications involving Honduran researchers and those from other countries. I was also involved managing funds so that this investigation was carried out in Honduras; I was responsible for applying for grants.”

**Figure 1:**
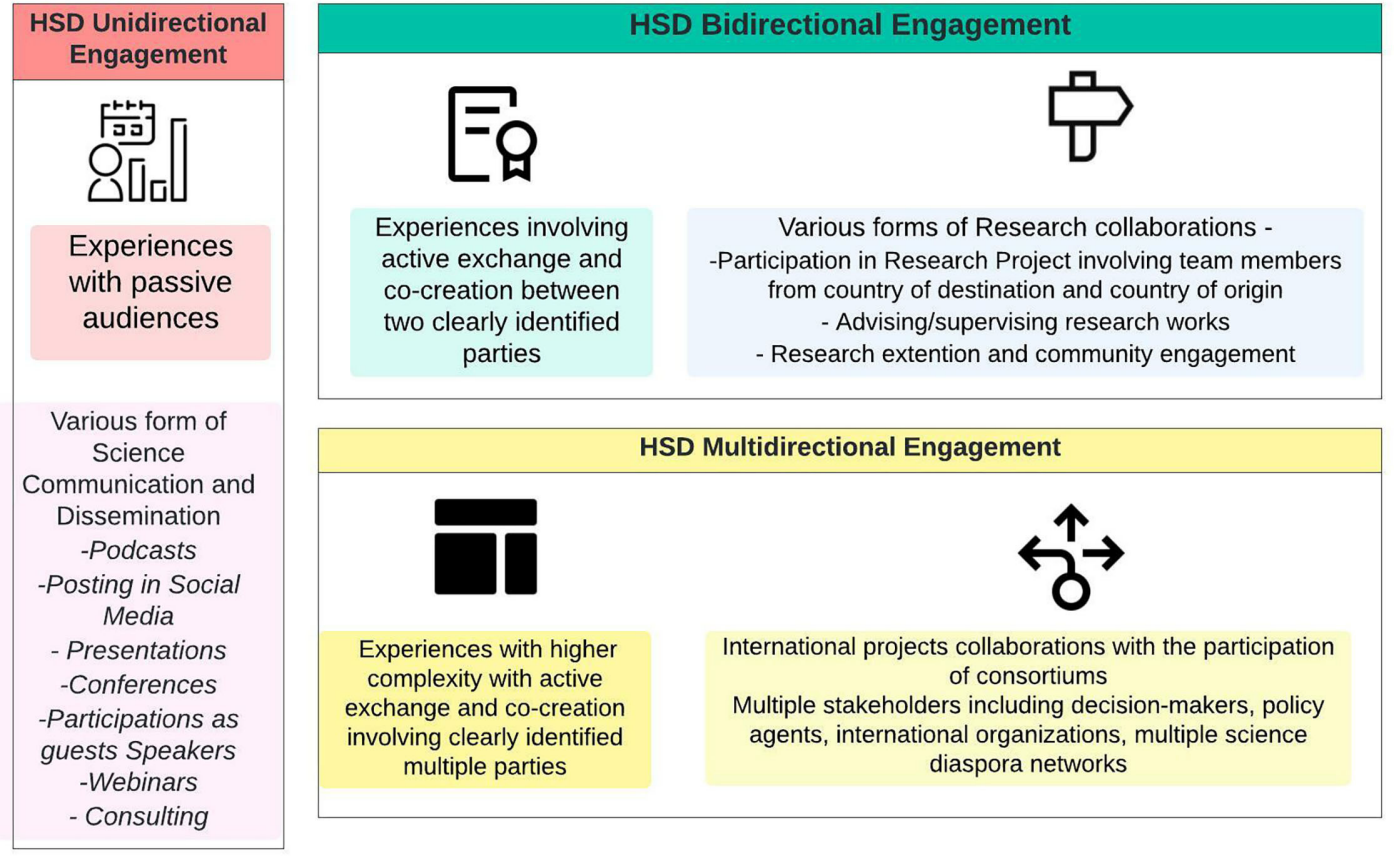
HSD directional engagement.

Figure 1 below illustrates the most recurrent examples of the engagement experienced by the participants.

## Conclusions

Concerning the notions about the scientific diaspora, the study participants showed little knowledge of the subject and, therefore, a lack of identification with the term. The most frequent association in the responses was with the brain drain. Likewise, there was a consensus on the need to leave the country due to the lack of scientific opportunities and networks. Most of the people interviewed indicated that their motivation for joining was for organizational purposes, to guarantee them common spaces with people from their areas of work, and to collaborate in the development and improvement of living conditions in Honduras. It is essential to highlight those members of the HSD who participated in this study expressed their commitment to the development of Honduras and the knowledge, expertise, and benefits of the networks acquired during their stay abroad. However, one of the barriers they found is the lack of interest, opportunities, and resources, including the government and the Honduran ST&I ecosystem. Due to the country's lack of interest in engaging them, three initiatives studied in this research have played a pivotal role in harnessing the scientific diaspora to create a model for collaboration that promotes the scientific development of the country and meets the global goals.

Findings provide evidence that in the case of HSD, the bottom-up approach has been explored and yields results. Individual initiatives have fostered the creation and consolidation of networks that have drawn attention and support from external actors in their evolution. An example of this has been Honduras Global. In the case of OWSD, Honduras's actions toward Honduran women scientists can be traced back to the construction of networks and communities by appealing to the imitation example going from part to a system. Yet, in this second case, the support of a global organization (OWSD Secretariat located in Trieste, Italy) has proven central to the sustainability of this network. Finally, the case of AGEAP-Zamorano incorporates the strong support of the institution. In terms of engagement for developing their country of origin, Honduras Global and OWSD Honduras show the most substantial emphasis on exerting the positive influence of their members to contribute to improving national S&T capacities and living conditions in Honduras. The case of AGEAP-Zamorano presents a solid focus on the career development of its members, having the effect of Honduras as a complementary goal.

We believe that this is the first step to delving into the topic of engaging the HSD. The contribution of this research is empirical. We present the first comprehensive analysis of experiences engaging the Honduras Scientific Diaspora for development. The study collects primary data from a new context such as Honduras and elaborates on the paradigm shift from the brain-drain approach, dominant in literature, to the new approach of Science Diplomacy and attention provided by the foreign policy to map the migration outflow of Honduran scientists. The emphasis is placed on the circular exchange and transnational mobility in the migration–development nexus.

## Data Availability Statement

Publicly available datasets were analyzed in this study. This data can be found here: http://hondurasglobal.org/, https://owsd.net/network/honduras, https://www.zamorano.edu/capitulos-nacionales/.

## Ethics Statement

The studies involving human participants were reviewed and approved by the Ethics Committee from the University of Technology of El Salvador (UTEC). All participants were asked to sign an electronic informed consent form prior to their participation.

## Author Contributions

KB took a leading role in conceptualization, data curation, project administration, methodology, resources, validation, and writing—original draft (lead). KA contributed to conceptualization, data curation, supervision, project administration, writing original draft, and supported visualization. RA-T involved in conceptualization (lead), data curation (equal), methodology (lead), writing original draft (equal), and visualization (support). SN involved in investigation (equal), visualization (supporting), writing original draft (equal), and writing—review and editing (equal). All authors contributed to the article and approved the submitted version.

## Conflict of Interest

The authors declare that the research was conducted in the absence of any commercial or financial relationships that could be construed as a potential conflict of interest.

## Publisher's Note

All claims expressed in this article are solely those of the authors and do not necessarily represent those of their affiliated organizations, or those of the publisher, the editors and the reviewers. Any product that may be evaluated in this article, or claim that may be made by its manufacturer, is not guaranteed or endorsed by the publisher.

## Footnotes

^1^ https://www.giz.de/en/worldwide/138.13.html

^2^ http://hondurasglobal.org/

^3^ https://owsd.net/network/honduras

^4^ https://www.zamorano.edu/capitulos-nacionales
